# Noninvasive Recording of True-to-Form Fetal ECG during the Third Trimester of Pregnancy

**DOI:** 10.1155/2014/285636

**Published:** 2014-10-08

**Authors:** Istvan Peterfi, Lorand Kellenyi, Andras Szilagyi

**Affiliations:** Department of Obstetrics and Gynecology, Kaposi Mór Teaching Hospital, Kaposvár, Hungary

## Abstract

*Objective*. The aim of the study was to develop a complex electrophysiological measurement system (hardware and software) which uses the methods of electrophysiology and provides significant information about the intrauterine status of the fetus, intending to obtain true-to-form, morphologically evaluated fetal ECG from transabdominal maternal lead. *Results*. The present method contains many novel ideas that allow creating true-to-form noninvasive fetal ECG in the third trimester of the pregnancy in 80% of the cases. Such ideas are the telemetric data collection, the “cleanse” of the real time recording from the maternal ECG, and the use of the cardiotocograph (CTG) that allows identifying the fetal heart events. The advantage of this developed system is that it does not require any qualified staff, because both the extraction of the information from the abdominal recording and the processing of the data are automatic. *Discussion*. Although the idea of a noninvasive fetal electrocardiography is more than 100 years old still there is no simple, effective, and cheap method available that would enable an extensive use. This developed system can be used in the third trimester of the pregnancy efficiently. It can produce true-to-form fetal ECGs with amplitude less than 10 *µ*V.

## 1. Introduction and Aim of the Study

Relatively few methods are available for assessing the intrauterine wellbeing of the fetus. The monitoring of the fetal heart rate (cardiotocography, CTG) is essential in the everyday obstetrical practice but the sensitivity and specificity of the method needs to be improved. The ultrasonographic blood flowmetry is a further option to assess fetal intrauterine condition, but it is only an indirect sign, such as the CTG. In case of pathological fetal heart rate the fetal scalp capillary blood pH is taken during delivery but since the method is invasive and technically difficult to carry out, a caesarean section is chosen in many cases. Fetal pulse oximetry during delivery is not used anymore. The magnetic cardiograph offers promising results but the expensive cost limits its spreading at the moment. The idea of fetal electrocardiogram (f-ECG) has been formulated over a century but the practical application is still pending in these days because of the technical difficulties. It is particularly difficult to record true-to-form ECG through the maternal abdomen in a noninvasive way which is fitting for the conformal morphological examinations in cardiology, although the direct fetal ECG during delivery with the help of a scalp electrode is available today for the everyday praxis [1].

The purpose of the study is to invent such a complex electrophysiological measurement system (hardware and software) that uses the methods of electrophysiology and is able to provide significant information about the intrauterine status of the fetus during pregnancy. Being aware of the fact that the fetal heart rate variations seen on CTG refer only indirectly to cellular damage, it is especially important to have direct access to fetal ECG. Retrospective reviews of cases concentrating on children with permanent neurological defects showed that the majority of them had no demonstrable hypoxic episodes during labour [2].

## 2. Method and Results

This system contains an* ad hoc *developed hardware and software. The hardware is composed of a preamplifier in the size of a cell phone that sends the sampled signals to the processing unit telemetrically. From here the data are transmitted to the computer through an A/D converter through several parallel channels simultaneously. The sampling passes on 2 kHz in a depth of 16 bits due to the according international practice [3] but this system is suitable to handle up to several orders of magnitude higher than sampling frequencies. The software was developed in Object Pascal language (in a Delphi developing environment) from the beginnings to aim this purpose.

The focus was on three main points during the developmental work: (1) to create true-to-form, morphologically evaluated fetal ECG from transabdominal maternal lead, (2) to record direct fetal ECG during the labour using fetal scalp electrode, and (3) to create fetal EEG registration and procession during delivery.

In this short introduction the first point will be detailed.

Several articles can be found about the issue using different—and difficult—mathematical models to solve the problem of indirect fetal ECG lead (with more or less success) [4], but a method which can be used in everyday practice has not been found yet. Papers on noninvasively recorded true-to-form fetal ECG with amplitude less than 30 *μ*V have not been published so far. Great advantage of the presented method is that it can be automatised and its application does not require high-qualified staff. In 70–80% of the cases it was possible to gain true-to-form, cardiologically evaluable fetal ECG.

In the course of this method only 3 standard Ag-AgCl electrodes were used: two active electrodes (positive and negative poles) and an active grounding. According to most of the studies one electrode is placed over the symphysis and the others are placed on the fundus of the uterus in the epigastrium to the left or to the right or a little lower according to the fetal rump. More than 70 measurements have been made with this system so far. Before recording the exact position, size of the fetus and the position of the placenta were ascertained with ultrasound. These experiences have shown that the largest fetal R wave can be accomplished when the active electrodes are perpendicular to the trunk of the fetus on the mother's abdominal wall. So in this position the electrodes are located accordingly to the electric axis of the fetal heart.

Numerous factors can cause complications during the extraction of the fetal signals from the record of the maternal transabdominal lead: the interference of the significantly higher amplitude of the maternal ECG signals, the contractions of the uterus, the maternal breathing, and the kinetic artefacts, not to mention the background electric noise presented in nonlaboratory environment.

In this noninvasive lead the amplitude of the maternal ECG signals falls within the range of 0.5 and 1.5 mV while the amplitude of the fetal ECG signals measured with standard Ag-AgCl electrodes through the mother's abdominal wall varies between 10 and 150 *μ*V. This corresponds to the EEG range but in this case the sampling rate used in EEG from 200 Hz to 2 kHz needs to be extended. From the higher sampling frequencies, f-ECG: 10–20 *μ*V versus EEG: 2–5 *μ*V, higher electric background noise is coming.

**Figure 1:**
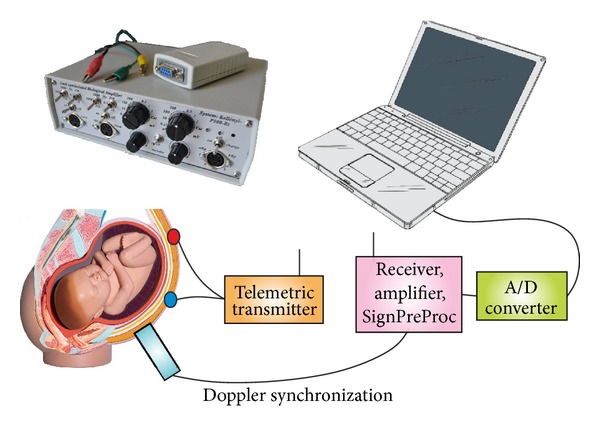
Schematic representation of the developed system. Standard Ag-AgCl electrodes are placed on the mother's abdomen. Preamplified signals are sent telemetrically to the receiver, where the first steps of signal processing happen. The collected data are transmitted through an A/D converter to the computer. Doppler signals are used to synchronize the fetal heart events.

The level of background noise power (50 Hz due to the power supply voltage noise primarily) can be reduced significantly by using radio telemetry technology [5]. The mother is related only to a battery-powered signal recording amplifier in a size of a cell phone operating in low voltage. The amplifier sends the signals to the signal processing unit telemetrically. Thus, this tool fully meets the human electrophysiological shock protection rules as well. The elementary procession of the information takes place in the signal processing unit. Then the data are transmitted to the computer through an A/D converter (Figure 1).

It is known that during linear averaging the amplitude of the useful signal loaded with statistical noise increases linearly with the number of averaging while the “noise” only increases with its square root. Although the maternal ECG waves are considered as noises on the abdominal recording in a strict mathematical sense, they cannot be considered to occur in a more or less well-defined periodicity.

To average the fetal signals the “cleanse” of the recording from the maternal ECG [6] is necessary. Ideally one could say that the cardiac cycles of the mother are morphologically identical. Of course, this is not valid because during respiration, the maternal heart rate varies substantially, as well as the maternal ECG amplitude. During recording one must strive to keep the mother in peace and make her breathe as shallow as possible. Women's heart rate in their second and third trimester of pregnancy is basically characterized by tachycardia. Independently from that, it is worth turning the pregnant woman slightly to the side so the significant tachycardia arising from the inferior vena cava compression can be reduced. Due to their size the R waves of the maternal ECG can easily be identified by both hardware and software (e.g., the derivation of the functions or just by the application of the level trigger or peak trigger). This system automatically identifies the maternal signals and after a definite (in case = 30) number of cycles it creates the cardiac cycles' average from the maternal ECG. Then it subtracts its own average from each detected maternal ECG waves in real time. With the aforementioned method one was able to “cleanse” the abdominal recording from maternal ECG significantly. The amplitude of the artefacts generated during the operation matches the size of the environmental noise.

**Figure 2:**
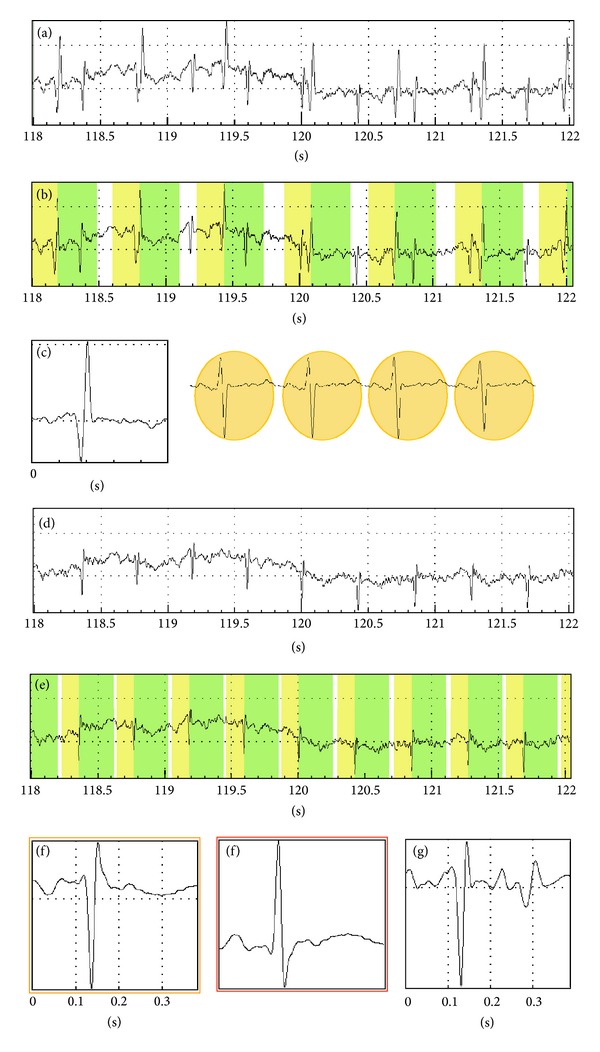
(a) Transabdominal record at the 39th week of pregnancy. The fetal ECG R wave is quite visible. (b) The software automatically identifies the maternal ECG R waves. (c) The system calculates the average of 30 maternal cardiac cycles. Then the computer extracts in real time the averaged signal from each identified maternal ECG heart event. (d) The “cleaned” abdominal signal, maternal waves are removed. (e) Using the same peak detection algorithm the fetal ECG R waves are identified. (f) After averaging 40 fetal heart events true-to-form fetal ECG is received. (g) It is very important to remove maternal waves, because otherwise the averaged fetal ECG cannot be assessed.

In a fortunate case (only 25–30% of the cases) the fetal R waves are clearly visible on the recording. If the fetus lies in cephalic presentation the maternal and fetal ECG R waves are opposite signs so the fetal signals can be identified relatively easily. In case of codirectional maternal and fetal R waves we can find and then average the fetal signals after “filtering” the maternal waves (Figure 2).

**Figure 3:**
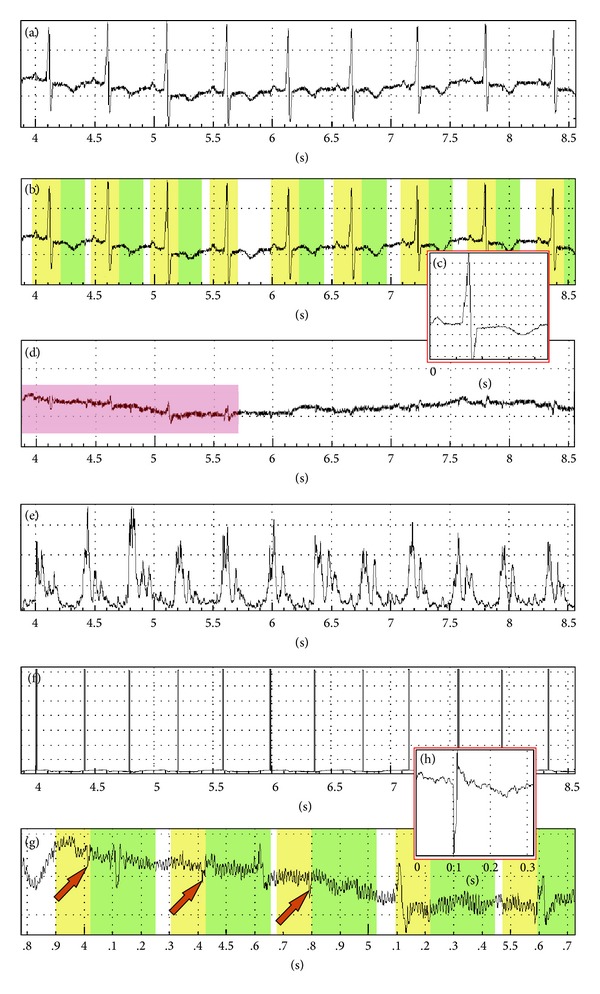
(a) Transabdominal record at the 34th week of pregnancy. Contrary to previous recordings, fetal signals are so small in this case that they cannot be identified. (b) The software automatically identifies the maternal ECG R waves. (c) The system calculates the average of 30 maternal cardiac cycles, and then the computer extracts in real time the averaged signal from each identified maternal ECG heart event. (d) The “cleaned” abdominal signal, maternal waves are removed. (e) The preprocessed sound signals of the cardiotocograph can be used to identify fetal cardiac events. (f) The hardware generates automatically trigger signs from the acoustic waves. (g) The identified fetal signals are averaged (in this case 76 fetal heart events are averaged). (h) The final result is an approximately true-to-form fetal ECG. It is important to emphasize that in the example shown above the fetal ECGs amplitude is less than 10 *μ*V.

Difficulties can occur if the fetal ECG's R waves measured through the abdominal wall cannot be detected because of the thickness of the maternal abdominal wall or other insulating layers (e.g., vernix caseosa) or simply because the pregnancy is in the early weeks of the third trimester. In other words if the amplitude of the fetal signal measured through the mother's abdominal wall is smaller than the environmental noise, in this case, one cannot identify fetal cardiac events, and so one cannot perform the averaging of multiple fetal cardiac cycles. To solve this problem the sound signals of the cardiotocograph can be used to identify fetal cardiac events. The heart (cardio) sensor is an ultrasonic sensor which works on the principle of the Doppler effect and beeps according to the flow (movement) degree. After proper procession the sounds can be used to produce signals. In order to obtain a signal corresponding to the purpose, the placement of a Doppler transducer is essential. If the ultrasound is reflected from the atrioventricular valve while the flow of the vena cava is not in the radius of the transducer, a sharp click can be heard so one can easily generate a trigger signal accordingly. This trigger signal does not necessarily coincide with the R wave of the fetal ECG, but it is in synchrony with it. The fetal shifts complicate the examination but if one manages to prepare a one-minute long recording while the fetus is at rest, then true-to-form fetal signals can be obtained with the averaging of approximately 120–160 heart events. By all means, the previous removal of the maternal signals is essential. With the method mentioned above the recording of 10 *μ*V or less true-to-form fetal ECG measured noninvasively through the mother's abdominal wall can be managed (Figure 3).

## 3. Conclusions and Remarks for the Future

The method and system described above are suitable for everyday use as the averaging and signal processing operations are made by the hardware and software automatically. The staff is only responsible for giving explanations and instructions to the mother and for the proper installation of the electrodes and the transducer on the mother's abdominal wall. The examination can be carried out for a small expense. Recording fetal ECG during the third trimester of the pregnancy may become an essential tool for the assessment of intrauterine fetal wellbeing, furthermore also for detecting fetal heart anomalies with ECG abnormalities. If one possesses a morphologically analysable fetal ECG, one is able to choose the place, manner, and time of the labour more thoroughly thereby the perinatal morbidity and mortality data can be improved. The device is also suitable for creating CTG curve during delivery through direct leads and to record direct fetal ECG in high quality. Experiments are designed in relation to the future measurement of fetal EEG [7].
